# Development and validation of a First Trimester Clinical Risk Score (FTRS) for predicting gestational diabetes mellitus in a low-resource setting: an observational cohort study

**DOI:** 10.1186/s13098-026-02257-2

**Published:** 2026-08-07

**Authors:** Sasmita Dash, Mohini, Manika Bose, Asima Das

**Affiliations:** 1 Department of Obstetrics and Gynaecology, IMS and SUM Hospital, Siksha ‘O’ Anusandhan (Deemed to be) University, Bhubaneswar, Odisha India; 2 Department of Obstetrics and Gynaecology, Kalinga Institute of Medical Sciences, KIIT University, Bhubaneswar, Odisha India; 3 Department of Pharmacology, IMS and SUM Hospital, Siksha ‘O’ Anusandhan (Deemed to be) University, Bhubaneswar, Odisha India

**Keywords:** Gestational diabetes mellitus, First-trimester clinical risk score, Clinical prediction model, Early screening, Risk prediction, Low-resource settings, Sensitivity and specificity

## Abstract

**Background:**

Several models have been developed for early prediction of gestational diabetes mellitus (GDM) during the first trimester; however, many rely on biochemical markers or are unsuitable for low-resource settings. This study aimed to develop and validate a simple first-trimester clinical risk score (FTRS) for early prediction of GDM using routinely available clinical variables.

**Methods:**

In this prospective cohort study, 730 pregnant women presenting before 14 weeks’ gestation at a tertiary care teaching hospital in Eastern India were enrolled. GDM was diagnosed using the Diabetes in Pregnancy Study Group India (DIPSI) criteria based on a 75-g oral glucose challenge test performed at three time points during pregnancy. Eight clinical predictors were evaluated using multivariable Poisson regression. Equal- weighted and weighted FTRS models were developed and assessed using receiver operating characteristic (ROC) curve analysis.

**Results:**

GDM developed in 271 (37.1%) participants. Eight clinical predictors were incorporated into the FTRS. The equal-weighted score (cut-off ≥ 3) achieved an area under the ROC curve (AUC) of 0.74, with 66.8% sensitivity and 82.9% specificity. The weighted score (cut-off ≥ 10) showed slightly better performance, with an AUC of 0.77, 70.2% sensitivity, and 83.4% specificity.

**Conclusion:**

The first-trimester clinical risk score (FTRS) is a simple, practical, and non-invasive tool for early prediction of GDM using routinely available clinical variables. Its ease of application makes it particularly suitable for early risk stratification and targeted screening in low-resource settings.

## Introduction

Gestational diabetes mellitus (GDM) is a common metabolic disorder of pregnancy, characterized by glucose intolerance first recognized during pregnancy [1]. It results from insulin resistance driven by placental hormones, leading to impaired glucose metabolism [1, 2]. GDM is associated with serious maternal and foetal complications, including miscarriage, polyhydramnios, preeclampsia, macrosomia, and a higher lifetime risk of type 2 diabetes [3–9]. The global burden of GDM is rising, particularly in developing countries [10]. In India, GDM affects approximately 10–14.3% of pregnancies, with some reports indicating a prevalence of over 30% in certain populations [11]. This is comparable to, or even higher than, that in high-income countries.

GDM is diagnosed using an oral glucose tolerance test (OGTT), either universally or selectively based on risk factors [12, 13]. In India, the Ministry of Health and Family Welfare (MoHFW) recommends universal screening for GDM at the first antenatal visit and repeat screening at 24–28 weeks of gestation if the initial test is negative.( MoHFW Technical & Operational Guidelines 2018) In resource-limited settings, the Diabetes in Pregnancy Study Group India (DIPSI) recommends a single-step 75-g oral glucose test performed irrespective of fasting status, which has been adopted in the national operational guidelines for GDM screening.( DIPSI Guidelines, Seshiah et al.)Unlike the conventional 50-g Glucose Challenge Test (GCT) used internationally as a screening test, the DIPSI strategy employs a 75-g oral glucose load and is used as a diagnostic test in the Indian healthcare system. The present study therefore aimed to develop a first-trimester clinical risk score for predicting GDM diagnosed according to the DIPSI criteria rather than to predict or validate a conventional GCT. However, practices vary across regions.

Despite national guidelines, implementation is often limited by resource constraints such as test availability, trained personnel, and follow-up mechanisms [9]. In low-resource settings, universal OGTT may not be feasible. Early identification of high-risk women using simple clinical factors could improve screening efficiency and reduce the burden on healthcare systems. Several models exist for predicting GDM, but many rely on biochemical markers or are not validated in the Indian population. A low-cost, clinical risk score applicable in the first trimester could help streamline GDM screening.

In this context, the present study was conducted to develop and validate a risk assessment scoring system for prediction of gestational diabetes mellitus in the first trimester based on multiple risk factors.

## Methods

### Study design and study setting

The current study was done as an observational analytical prospective cohort study conducted in a tertiary care teaching hospital of Eastern India.

## Study period and study population

The study was conducted from May 2023 to May 2025, in the Department of Obstetrics and Gynaecology. Study population included pregnant women attending the antenatal outpatient department (OPD) during their first trimester of pregnancy, and were screened for eligibility and recruited into the study.

## Inclusion criteria

- Pregnant women with a singleton pregnancy attending the antenatal outpatient department.
- Gestational age ≤ 14 weeks completed at the time of enrolment, confirmed by the last menstrual period and/or first-trimester ultrasonography.
- Age ≥ 18 years or older.
- Women without a previous diagnosis of diabetes mellitus.

## Exclusion criteria

- Pre-existing diabetes mellitus (Type 1 or Type 2) or overt diabetes diagnosed at the booking visit.
- Pregnancies conceived through assisted reproductive techniques (ART), if intended to be excluded because of their different baseline risk profile.
- Chronic medical disorders known to influence glucose metabolism (e.g., chronic corticosteroid therapy, Cushing’s syndrome) or severe systemic illness.
- Incomplete baseline clinical data or loss to follow-up before assessment for gestational diabetes mellitus.

## Sample size

Sample size calculation for discovery cohort: To study 8 known risks, at least 120 patients would be required for regression analysis. Assuming the prevalence of GDM in pregnant women to be 8–10%, we will need to follow up at least 730 pregnant women. The incidence of GDM was calculated with 95% confidence interval (CI).

### Study variables

The outcome variable was GDM. Gestational diabetes mellitus was diagnosed using the Diabetes in Pregnancy Study Group India (DIPSI) single-step 75-g oral glucose test, performed irrespective of fasting status, according to the recommendations of the Ministry of Health and Family Welfare, Government of India. Women underwent testing at enrolment (< 14 weeks), and those testing negative were rescreened at 24–28 weeks and again at 32–36 weeks of gestation. A 2-hour plasma glucose value ≥ 140 mg/dL after the 75-g oral glucose load was considered diagnostic of GDM according to DIPSI criteria.

In our study protocol, all enrolled women underwent glucose testing at booking. Women who tested negative were rescreened at 24–28 weeks of gestation, and those remaining negative underwent a final test at 32–36 weeks, in accordance with the national screening recommendations.

The following variables, measured in the first trimester, were considered as predictor variables in the model. Age was dichotomized as > 30 years and ≤ 30 years, based on established risk thresholds for GDM. Body weight and height were measured at the first antenatal visit (< 14 weeks of gestation) using calibrated equipment by trained healthcare personnel. BMI was calculated from these measured values and categorized according to the Asia-Pacific. Place of residence was classified as urban or rural according to the Census of India administrative classification based on the participant’s usual place of residence at enrolment. History of PCOS was obtained from previous medical records or a documented diagnosis by a treating obstetrician/gynaecologist**. **History of Macrosomia was defined as the delivery of a previous infant weighing > 4 kg as documented from the prior pregnancy documentations. A family history of DM was defined as having at least one first-degree relative with type 2 diabetes. History of smoking was elicited by self-reported history of tobacco smoking prior to or during pregnancy in last one year of the current pregnancy. Blood pressure was measured by trained healthcare providers during the first antenatal visit using a standardized procedure in accordance with established guidelines. Measurements were taken by a manual sphygmomanometer on the right arm, with the participant in a seated position after at least five minutes of rest. Two readings were taken five minutes apart, and the average of the two readings was recorded. If the initial two readings varied by more than 10 mmHg, a third reading was taken, and the readings were averaged. Diastolic blood pressure (DBP) was defined as the fifth Korotkoff sound. For this study, DBP was categorized as high (≥ 90 mmHg) or normal (< 90 mmHg).

### Statistical analysis

Baseline characteristics were collected from the participants through predesigned data collection form by the investigators at the first antenatal visit in the first trimester.

The data was collected by the investigators in a dedicated place at the outpatient department (OP). Upon obtaining informed consent, participants were administered a structured questionnaire capturing the baseline status of the predictor variables. These participants were followed up till term for the GCT values- at first visit, at 28 weeks and at 36 weeks. The values were noted in the data collection form. Standard care was provided upon diagnosis of GDM as per the hospital protocol.

Univariate analysis was first performed using Poisson regression with robust standard errors to estimate unadjusted relative risks (RRs) and their 95% confidence intervals (CIs). Variables having a p value < 0.2 were included in the multivariate model. Multicollinearity was assessed using variance inflation factors (VIF), and variables with VIF > 5 were considered for exclusion, although no such cases were found. A multivariate Poisson regression with robust error variance was conducted to estimate adjusted RRs (aRR) for the final set of predictors. Based on this model, two scoring systems were developed to predict the risk of GDM in the first trimester. The first was equal-weighted score where each risk factors were assigned a score of 1 if present, and 0 if absent, resulting in a total score ranging from 0 to 8. The second was weighted score where regression coefficients (β) from the final multivariate model were scaled by dividing each coefficient by the smallest β, then rounded to the nearest whole number to assign weights to each predictor. The final weighted score was calculated as the sum of these weighted values.

Receiver Operating Characteristic (ROC) curve analysis was conducted for both scoring systems to evaluate diagnostic performance. Area Under the Curve (AUC) was estimated for each model. The optimal cut-off point for the weighted score was identified using Youden’s Index (sensitivity + specificity – 1), and diagnostic metrics including sensitivity, specificity, positive predictive value (PPV), negative predictive value (NPV), and overall accuracy were calculated. A two-sided p-value < 0.05 was considered statistically significant for all statistical tests.

Data were entered in Microsoft Excel and analysed in Stata version 18.0. Descriptive statistics were used to summarize the distribution of baseline characteristics. Categorical predictors were described using frequencies and percentages and continuous variables were presented as mean and standard deviation (SD). The outcome variable was the occurrence of gestational diabetes mellitus (GDM), coded as a binary outcome.

## Results

We recruited 730 patients in the study who completed all the follow-ups. The mean age of the participants was 29.2 years (SD 4.7 years) with an age range of 18 and 47 years. Majority of participants were < 30 years (*n* = 459, 62.88%) as shown in Table 1. A total of 271 (37.12%; 95% CI: 33.54 to 40.7%) women developed GDM.

**Table 1:** Clinical characteristics of the participants

Variables		Frequency (%)
Age, years	< 30 years	459 (62.88)
	>=30 years	271 (37.12)
BMI, kg/M^2^	< 23 kg/M^2^	523 (71.64)
	>=23 kg/M^2^	207 (28.36)
Gravida	Multi	375 (51.44)
	Primi	354 (48.56)
Area of residence	Rural	328 (44.93)
	Urban	402 (55.07)
Religion	Christian	9 (1.23)
	Hindu	708 (96.99)
	Muslim	13 (1.78)
SES*	Lower	10 (1.37)
	Lower middle	407 (55.75)
	Upper middle	286 (39.18)
	Upper	27 (3.7)

*SES- Socio-economic status, based on Kuppuswamy Socio-economic scale, 2023

Predictors of GDM: Univariate analysis suggests that all the variables depicted in Table 2, are associated with high risk of GDM development. The adjusted relative risks associated with the variables are as follows- age > = 30 (aRR 1.16; 95% CI: 1.0-1.38), BMI > = 23 Kg/m^2^ (aRR 1.5; 95% CI: 1.01–1.62), urban residence (aRR 3.25; 95% CI: 2.46–4.31), history of PCOS (aRR 1.8; 95% CI: 1.47–2.2), prior history of macrosomia (aRR 1.94; 95% CI: 1.51–2.48), family history of diabetes (aRR 1.76; 95% CI: 1.48–2.1), history of smoking (aRR 1.67; 95% CI: 1.36–2.06), and high DBP ( > = 90 mm of hg) recorded during the current pregnancy (aRR 1.73; 95% CI: 1.41–2.12).

**Table 2:** Regression analysis for GDM predictors

Variables	Relative risk (RR), unadjusted, 95% CI	Relative risk (RR), adjusted, (95% CI)	*p*-value
Age > = 30 years	1.56 (1.3–1.88)	1.16 (1.0-1.38)	0.07*
BMI > = 23 Kg/m^2^	1.27 (1.07–1.58)	1.5 (1.01–1.62)	0.048*
Urban residence	4.1 (3.08–5.45)	3.25 (2.46–4.31)	< 0.001*
H/O PCOS	1.76 (1.43–2.16)	1.8 (1.47–2.2)	< 0.001*
H/O macrosomia	1.56 (1.28–1.88)	1.94 (1.51–2.48)	< 0.001*
Family h/o DM	2.06 (1.72–2.46)	1.76 (1.48–2.1)	< 0.001*
H/O smoking	1.77 (1.4–2.24)	1.67 (1.36–2.06)	< 0.001*
High DBP ( > = 90 mm of hg)	2.0 (1.65–2.41)	1.73 (1.41–2.12)	< 0.001*

**Fig. 1:**
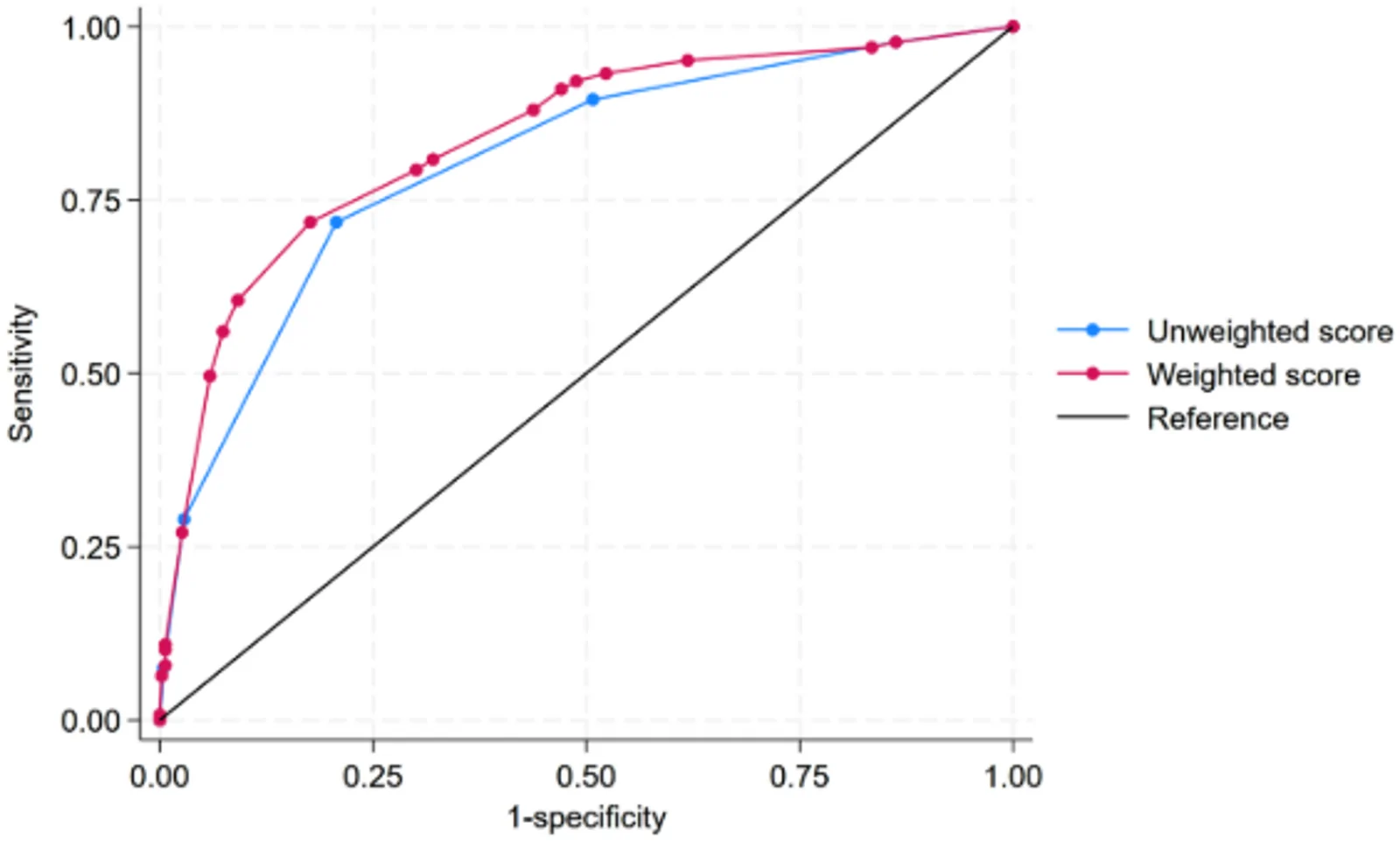
ROC curves comparing the diagnostic performance of equal-weighted and weighted first-trimester risk scores for predicting GDM

Composite score: Presence of each of the above variables were considered a score of 1 and absence was assigned a score of 0. Adding score of all the variables mentioned in Table 2, a maximum score of eight and a minimum score of 0 were possible. After adding, we found that the maximum score was five for a participant. Based on the ROC curve (Fig. 1), a cut-off score of 3 or more was considered as higher chance of developing GDM. The sensitivity of this cut-off was 66.8%% (95% CI: 61% to 72.2%) and specificity was 82.9% (95% CI: 79.1 to 86.3%) with a high area under the curve (AUC: 0.74; 95% CI: 0.71 to 0.78).

**Table 3:** Calculating weighted score

Variable	Co-efficient (β)	β ÷ 0.16	Rounded Weight
Age > = 30 years	0.16	1.00	1
BMI > = 23 Kg/m^2^	0.24	1.50	2
Urban residence	1.18	7.38	7
H/O PCOS	0.59	3.69	4
H/O macrosomia	0.66	4.13	4
Family h/o DM	0.57	3.56	4
H/O smoking	0.51	3.19	3
High DBP ( > = 90 mm of HPg)	0.57	3.56	4

BMI- Body mass Index; H/O-history of, DM-diabetes mellitus, ;DBP-diastolic blood pressure; PCOs- polycystic ovarian syndrome

We also calculated the weighted score after considering the beta coefficients of the variables from the regression model and dividing the values with the smallest beta and rounding off the values to the next integer vide- methods, shown in Table 3. The minimum and maximum score was 1 and 29- more the score, higher is the risk. After constructing the ROC curve (Fig. 1), the cut-off was calculated as 10. Based on this cut-off, we noticed a marginal improvement in all the diagnostic test characteristics as shown in Table 4. The sensitivity was calculated to be 70.2% (95% CI: 64.4–75.6%), which was higher than the sensitivity based on the unweighted scoring; however, the difference (3.4%; 95% CI: -1.36% to 8.16%, *p* = 0.16) was not statistically significant.

**Table 4:** Diagnostic test accuracy for the composite score cut-off of three

Diagnostic test parameter	Unweighted Estimate (95% CI)	Weighted estimate (95% CI)
Sensitivity	66.8% (61.0-71.2%)	70.2% (64.4–75.6%)
Specificity	82.9% (79.1–86.3%)	83.4% (79.7–86.7%)
ROC area	0.74 (0.71–0.78)	0.77 (0.74–0.8)
Likelihood ratio (+)	3.91 (3.13–4.88)	4.24 (3.4–5.29)
Likelihood ratio (-)	0.4 (0.34–0.48)	0.36 (0.3–0.43)
Positive predictive value	71.8% (66.0-77.1%)	71.8% (66.0-77.1%)
Negative predictive value	79.3% (75.3–82.9%)	82.4% (78.6–85.7%)

## Discussion

The present study developed and validated a simple, clinic-based First Trimester Risk Score (FTRS) for early prediction of gestational diabetes mellitus (GDM) using routinely available antenatal variables in an Indian population. The score was derived from eight binary clinical predictors and evaluated in both equal-weighted and weighted formats. Both models demonstrated satisfactory predictive ability, while the weighted score showed marginally superior diagnostic performance. The findings suggest that the FTRS may serve as a feasible and cost-effective tool for early identification of women at high risk for GDM, particularly in resource-constrained healthcare settings.

Globally, several prediction models have been proposed for early detection of GDM, with varying combinations of maternal characteristics, anthropometric parameters, and biochemical markers. Models developed by Syngelaki et al. [14] and Teede et al. [15] primarily relied on clinical variables such as maternal age, BMI, ethnicity, and obstetric history, whereas Nanda et al. [16] improved predictive accuracy by incorporating biomarkers including adiponectin and sex hormone-binding globulin (SHBG). Although biomarker-based models generally demonstrate higher predictive precision, their applicability in low- and middle-income countries remains limited due to increased cost, laboratory dependency, and reduced accessibility. In contrast, our study intentionally focused on easily obtainable clinical variables, making the proposed score practical for routine antenatal screening even at peripheral healthcare facilities. This is particularly relevant in the Indian context, where barriers such as limited availability of OGTT, out-of-pocket healthcare expenditure, and inadequate follow-up continue to impede timely diagnosis and management of GDM [17].

Maternal age and obesity are among the most consistently reported predictors of GDM across different populations [15, 16, 18–20]. Similar to previous studies, we observed that maternal age ≥ 30 years and BMI ≥ 23 kg/m² were significantly associated with increased risk of GDM. The lower BMI threshold used in our study reflects Asian Indian-specific recommendations, recognizing the higher metabolic risk among South Asians even at relatively lower BMI values. This finding aligns with earlier Indian studies that demonstrated increased insulin resistance and adiposity-related metabolic dysfunction at lower BMI cut-offs compared with Western populations.

In addition to conventional predictors, our study identified urban residence and elevated diastolic blood pressure as strong independent predictors of GDM. Urban residence demonstrated one of the highest relative risks in our cohort, possibly reflecting urban lifestyle-related factors such as sedentary behaviour, dietary transitions, and increased prevalence of obesity and metabolic syndrome. While urbanization has been linked to rising diabetes prevalence in previous epidemiological studies, it has not been routinely incorporated into most clinical prediction models. Similarly, elevated diastolic blood pressure showed a strong association with GDM risk, suggesting a possible shared pathophysiological basis involving endothelial dysfunction, insulin resistance, and cardiometabolic abnormalities. This observation supports findings from earlier studies that reported a relationship between early pregnancy blood pressure and subsequent glucose intolerance, although blood pressure parameters are less frequently included in existing prediction tools.

Smoking history and polycystic ovarian syndrome (PCOS) also emerged as important predictors in the present study. PCOS is a well-recognized risk factor for insulin resistance and future diabetes, and its association with GDM has been consistently reported in earlier literature [19, 20]. However, smoking history is often underreported or excluded from prediction models despite evidence linking smoking to chronic inflammation, oxidative stress, and impaired glucose metabolism. Inclusion of these factors may therefore improve the comprehensiveness of risk assessment in diverse populations. Furthermore, family history of diabetes mellitus and previous history of macrosomia were significantly associated with GDM in our cohort, findings that are in agreement with studies by Nanda et al., Syngelaki et al., and van Leeuwen et al. [15, 16, 21]. These variables likely reflect underlying genetic susceptibility and pre-existing metabolic risk.

When compared with previously published prediction models, the diagnostic performance of our weighted score was comparable despite relying exclusively on clinical variables. A recent external validation study evaluating multiple clinical prediction models, including those developed by Nanda, Syngelaki, Teede, van Leeuwen, and Caliskan, reported AUC values ranging from 0.68 to 0.75 [22]. Biomarker-based models incorporating adiponectin and SHBG demonstrated AUCs close to 0.78, with sensitivity and specificity generally ranging between 65% and 78% [23]. In the present study, the weighted FTRS achieved an AUC of 0.77 with sensitivity above 70% and specificity exceeding 80%, indicating comparable discriminatory performance. Unlike biomarker-based approaches, the present model predicts gestational diabetes mellitus diagnosed using the DIPSI screening strategy recommended in India and does not require additional laboratory biomarkers or specialized assays. The high specificity observed in our model may also help reduce unnecessary OGTT testing among low-risk women, thereby optimizing healthcare resources.

The practical utility of the present score lies in its simplicity and potential applicability during the first antenatal visit. Early identification of women at increased risk for GDM could facilitate targeted lifestyle interventions, closer glycemic surveillance, and timely diagnostic testing. Since most predictors included in the model are routinely documented during antenatal assessment, the score can be easily integrated into existing maternal healthcare programs without additional financial burden or infrastructural requirements. This is particularly important in developing countries, where universal biochemical screening during early pregnancy may not always be feasible.Nevertheless, the prospective design, standardized data collection, and use of clinically relevant variables strengthen the internal validity of the study findings.

Overall, the present study demonstrates that a simple clinical risk score based on first trimester characteristics can provide reasonably accurate prediction of GDM without reliance on expensive biochemical investigations. The FTRS may therefore represent a practical, scalable, and resource-sensitive approach for early GDM risk stratification in the Indian healthcare setting.

### Limitations

Despite these strengths, the study has certain limitations. Being a hospital-based study, selection bias cannot be excluded. The diagnosis of GDM was based on the DIPSI criteria recommended in India, which differ from ADA and IADPSG diagnostic criteria and may limit international comparability. In addition, dietary intake, physical activity, socioeconomic determinants, and psychosocial factors were not evaluated. External validation in geographically diverse populations is required before widespread implementation of the proposed First Trimester Risk Score.

## Conclusion

FTRS is a simple, first-trimester clinical risk score for predicting gestational diabetes mellitus using eight routinely available variables. Both weighted scoring model demonstrated strong discriminatory performance, with an AUC of 0.88 and good sensitivity and specificity. Both the weighted and unweighted scoring models demonstrated satisfactory predictive performance for identifying women likely to develop GDM diagnosed according to the DIPSI criteria. Owing to its simplicity, the unweighted score may be more suitable for routine use in low-resource healthcare settings.

## Data Availability

No datasets were generated or analysed during the current study.

## References

[1] Cunningham FG, Leveno KJ, Dashe J, Ho man B, Spong C, Casey BM. Williams Obstetrics, 2022.

[2] Nakshine VS, Jogdand SD. A comprehensive review of gestational diabetes mellitus: impacts on maternal health, fetal development, childhood outcomes, and long-term treatment strategies. Cureus. 2023;15(10). 10.7759/cureus.47500. PMC10663705 38021940

[3] Casey BM, Lucas MJ, McIntire DD, Leveno KJ. Pregnancy outcomes in women with gestational diabetes compared with the general obstetric population. Obstet Gynecol. 1997;90(6):869–73. 10.1016/S0029-7844(97)00542-5. 9397092

[4] Crowther CA, Hiller JE, Moss JR, McPhee AJ, Jeffries WS, Robinson JS. Effect of treatment of gestational diabetes mellitus on pregnancy outcomes. N Engl J Med. 2005;352(24):2477–86. 10.1056/NEJMoa042973. 15951574

[5] Clausen TD, Mathiesen ER, Hansen T, Pedersen O, Jensen DM, Lauenborg J, Damm P. High prevalence of type 2 diabetes and pre-diabetes in adult offspring of women with gestational diabetes mellitus or type 1 diabetes: the role of intrauterine hyperglycemia. Diabetes Care. 2008;31(2):340–6. 10.2337/dc07-1596. 18000174

[6] Metzger BE, Lowe LP, Dyer AR, Trimble ER, Sheridan B, Hod M, Chen R, Yogev Y, Coustan DR, Catalano PM, Giles W. Hyperglycemia and Adverse Pregnancy Outcome (HAPO) Study: associations with neonatal anthropometrics. Diabetes. 2009;58(2):453. https://www.proquest.com/scholarly-journals/hyperglycemia-adverse-pregnancy-outcome-hapo/docview/216480264/se-2?accountid=131615. 10.2337/db08-1112 PMC2628620 19011170

[7] Feig DS, Zinman B, Wang X, Hux JE. Risk of development of diabetes mellitus after diagnosis of gestational diabetes. CMAJ. 2008;179(3):229–34. 10.1503/cmaj.080012. PMC2474881 18663202

[8] Bellamy L, Casas JP, Hingorani AD, Williams D. Type 2 diabetes mellitus after gestational diabetes: a systematic review and meta-analysis. lancet. 2009;373(9677):1773–9. 10.1016/S0140-6736(09)60731-5 19465232

[9] Egan AM, Dow ML, Vella A. A review of the pathophysiology and management of diabetes in pregnancy. InMayo Clinic Proceedings 2020 Dec 1 (Vol. 95, No. 12, pp. 2734–2746). Elsevier. 10.1016/j.mayocp.2020.02.019 32736942

[10] Wang H, Li N, Chivese T, Werfalli M, Sun H, Yuen L, Hoegfeldt CA, Powe CE, Immanuel J, Karuranga S, Divakar H. IDF diabetes atlas: estimation of global and regional gestational diabetes mellitus prevalence for 2021 by international association of diabetes in pregnancy study group’s criteria. Diabetes Res Clin Pract. 2022;183:109050. 10.1016/j.diabres.2021.109050. 34883186

[11] Mantri N, Goel AD, Patel M, Baskaran P, Dutta G, Gupta MK, Yadav V, Mittal M, Shekhar S, Bhardwaj P. National and regional prevalence of gestational diabetes mellitus in India: a systematic review and Meta-analysis. BMC Public Health. 2024;24(1):527. 10.1186/s12889-024-18024-9. PMC10877914 38378536

[12] Metzger BE, Gabbe SG, Persson B, Lowe LP, Dyer AR, Oats JJ, Buchanan TA. International association of diabetes and pregnancy study groups recommendations on the diagnosis and classification of hyperglycemia in pregnancy: response to Weinert. Diabetes Care. 2010;33(7):e98. 10.2337/dc10-0719. PMC2827530 20190296

[13] Walker JD. NICE guidance on diabetes in pregnancy: management of diabetes and its complications from preconception to the postnatal period. NICE clinical guideline 63. London, March 2008. Diabet Med. 2008;25(9):1025–7. 10.1111/j.1464-5491.2008.02532.x. 19183306

[14] Syngelaki A, Pastides A, Kotecha R, Wright A, Akolekar R, Nicolaides KH. First-trimester screening for gestational diabetes mellitus based on maternal characteristics and history. Fetal Diagn Ther. 2015;38(1):14–21. 10.1159/000369970. 25531073

[15] Teede HJ, Harrison CL, Teh WT, Paul E, Allan CA. Gestational diabetes: development of an early risk prediction tool to facilitate opportunities for prevention. Aust N Z J Obstet Gynaecol. 2011;51(6):499–504. 10.1002/pd.2636. 21951203

[16] Nanda S, Savvidou M, Syngelaki A, Akolekar R, Nicolaides KH. Prediction of gestational diabetes mellitus by maternal factors and biomarkers at 11 to 13 weeks. Prenat Diagn. 2011;31(2):135–41. 10.1111/j.1479-828X.2011.01356.x. 21268030

[17] Morampudi S, Balasubramanian G, Gowda A, Zomorodi B, Patil AS. The challenges and recommendations for gestational diabetes mellitus care in India: A review. Front Endocrinol. 2017;8:56. 10.3389/fendo.2017.00056. PMC5364143 28392778

[18] Naylor CD, Sermer M, Chen E, Farine D. Selective screening for gestational diabetes mellitus. N Engl J Med. 1997;337(22):1591–6. 10.1056/NEJM199711273372204. 9371855

[19] Caliskan E, Kayikcioglu F, Öztürk N, Koc S, Haberal A. A population-based risk factor scoring will decrease unnecessary testing for the diagnosis of gestational diabetes mellitus. Acta Obstet Gynecol Scand. 2004;83(6):524–30. 10.1080/j.0001-6349.2004.00389.x. https://www.tandfonline.com/doi/abs/. 15144332

[20] Van Leeuwen M, Opmeer BC, Zweers EJ, Van Ballegooie E, Ter Brugge HG, De Valk HW, Visser GH, Mol BW. Estimating the risk of gestational diabetes mellitus: a clinical prediction model based on patient characteristics and medical history. BJOG: Int J Obstet Gynecol. 2010;117(1):69–75. 10.1111/j.1471-0528.2009.02425.x. 20002371

[21] Zhang Y, Xiao CM, Zhang Y, Chen Q, Zhang XQ, Li XF, Shao RY, Gao YM. Factors associated with gestational diabetes mellitus: a Meta-analysis. J Diabetes Res. 2021;2021(1):6692695. 10.1155/2021/6692695. PMC8128547 34046504

[22] Meertens LJ, Scheepers HC, van Kuijk SM, Roeleveld N, Aardenburg R, van Dooren IM, Langenveld J, Zwaan IM, Spaanderman ME, van Gelder MM, Smits LJ. External validation and clinical utility of prognostic prediction models for gestational diabetes mellitus: a prospective cohort study. Acta Obstet Gynecol Scand. 2020;99(7):891–900. 10.1111/aogs.13811. PMC7317858 31955406

[23] Iliodromiti S, Sassarini J, Kelsey TW, Lindsay RS, Sattar N, Nelson SM. Accuracy of circulating adiponectin for predicting gestational diabetes: a systematic review and meta-analysis. Diabetologia. 2016;59(4):692–9. 10.1007/s00125-015-3855-6 PMC4779132 26768001

